# Specific effect of hypobaria on cerebrovascular hypercapnic responses in hypoxia

**DOI:** 10.14814/phy2.14372

**Published:** 2020-02-25

**Authors:** Mathias R. Aebi, Nicolas Bourdillon, Andres Kunz, Denis Bron, Grégoire P. Millet

**Affiliations:** ^1^ Institute of Sport Sciences University of Lausanne Lausanne Switzerland; ^2^ Aeromedical Center (AeMC) Swiss Air Force Dübendorf Switzerland; ^3^ Becare SA Renens Switzerland

**Keywords:** cerebral blood flow autoregulation, cerebral oxygen delivery, hypobaria, hypoxia

## Abstract

It remains unknown whether hypobaria plays a role on cerebrovascular reactivity to CO_2_ (CVR). The present study evaluated the putative effect of hypobaria on CVR and its influence on cerebral oxygen delivery (cDO_2_) in five randomized conditions (i.e., normobaric normoxia, NN, altitude level of 440 m; hypobaric hypoxia, HH at altitude levels of 3,000 m and 5,500 m; normobaric hypoxia, NH, altitude simulation of 5,500 m; and hypobaric normoxia, HN). CVR was assessed in nine healthy participants (either students in aviation or pilots) during a hypercapnic test (i.e., 5% CO_2_). We obtained CVR by plotting middle cerebral artery velocity versus end‐tidal CO_2_ pressure (P_ET_CO_2_) using a sigmoid model. Hypobaria induced an increased slope in HH (0.66 ± 0.33) compared to NH (0.35 ± 0.19) with a trend in HN (0.46 ± 0.12) compared to NN (0.23 ± 0.12, *p* = .069). P_ET_CO_2_ was decreased (22.3 ± 2.4 vs. 34.5 ± 2.8 mmHg and 19.9 ± 1.3 vs. 30.8 ± 2.2 mmHg, for HN vs. NN and HH vs. NH, respectively, *p* < .05) in hypobaric conditions when compared to normobaric conditions with comparable inspired oxygen pressure (141 ± 1 vs. 133 ± 3 mmHg and 74 ± 1 vs. 70 ± 2 mmHg, for NN vs. HN and NH vs. HH, respectively) During hypercapnia, cDO_2_ was decreased in 5,500 m HH (*p* = .046), but maintained in NH when compared to NN. To conclude, CVR seems more sensitive (i.e., slope increase) in hypobaric than in normobaric conditions. Moreover, hypobaria potentially affected vasodilation reserve (i.e., MCAv autoregulation) and brain oxygen delivery during hypercapnia. These results are relevant for populations (i.e., aviation pilots; high‐altitude residents as miners; mountaineers) occasionally exposed to hypobaric normoxia.

## Graphical abstract

The present study was the first one to compare cerebrovascular CO_2_ reactivity during acute exposure in various normobaric/hypobaric and normoxic/hypoxic conditions. Our results suggest that vascular reserve to dilate may be blunted in hypobaria, either in hypoxic or normoxic conditions, since midpoint was left shifted. This blunt effect in hypobaria could impair cerebral oxygen delivery.

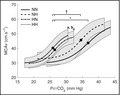

## 1 INTRODUCTION

Cerebral blood flow (CBF) regulation is very sensitive to hypoxia and regulates the cerebral oxygen delivery (cDO_2_) maintenance. CBF is regulated by complex vasoactive responses of the middle cerebral artery (MCA) (Imray et al., 2014; Willie, Smith, et al., 2014; Willie, Smith, Tzeng, Fisher, & Ainslie, 2014), extracranial cerebral vessels (Lewis, Messinger, Monteleone, & Ainslie, 2014) and in the pial mater arterioles (Wolff, 1930). There is a complex effect of oxygen arterial pressure (PaO_2_) and carbon dioxide arterial pressure (PaCO_2_) on CBF. More precisely, CBF is lowered by around 3%–4% for each mmHg of PaCO_2_ decrease (Ainslie & Duffin, 2009; Brugniaux, Hodges, Hanly, & Poulin, 2007; Willie et al., 2012). On the contrary, increases in PaCO_2_ and in blood pH are major factors increasing CBF via a common pathway, due to their vasoactive effects (Willie, Smith, et al., 2014; Willie, Tzeng, et al., 2014). When exposed to acute hypoxia (from minutes to hours), cerebral vasodilatation (i.e., increase in MCA diameter) occurs to limit the cDO_2_ decrease (Imray et al., 2014; Mikhail Kellawan, Harrell, Roldan‐Alzate, Wieben, & Schrage, 2017; Wilson et al., 2011). This regulation leads to an increase in cerebral oxygen delivery by 0.5%–2.5% of SaO_2_ decrease (Cohen, Alexander, Smith, Reivich, & Wollman, 1967; Jensen, Sperling, Severinghaus, & Lassen, 1996; Willie et al., 2012). On the other hand, hypoxia‐induced hyperventilation and hypocapnia result in a vasoconstrictor stimulus, but vasodilation typically prevails as consistent increase in CBF were observed at altitude, despite hypocapnia (Willie, Smith, et al., 2014; Willie, Tzeng, et al., 2014). There are several studies demonstrating the compensatory rise in CBF upon acute exposure to isocapnic hypoxia to maintain cDO_2_ (for review see, Hoiland, Bain, Rieger, Bailey, and Ainslie (2016). cDO_2_ in acute hypoxia is thus related to cerebral vasodilation, which compensates the hypocapnic vasoconstriction induced by chemoreflex‐driven ventilation (Teppema & Dahan, 2010).

Although still debated, hypobaric hypoxia (HH) may be more severe than normobaric hypoxia (NH) at a given inspired oxygen pressure (Millet, Faiss, & Pialoux, 2012). As an example, HH induces greater hypocapnia and blood alkalosis when compared to NH during acute exposure, which may be the consequence of an increase in ventilatory dead space (Savourey, Launay, Besnard, Guinet, & Travers, 2003). These differences between NH and HH may therefore induce changes in the cerebrovascular regulation.

One of the ways to assess how the cerebral vasculature regulates CBF is through measuring reactivity to CO_2 _(CVR) and can be measured by the blood velocity in the middle cerebral artery (Ainslie & Ogoh, 2010). CVR is regulated by hydrogen ion concentration (i.e., pH). At altitude, with changes in acid–base status, the relationship between changes in P_a_CO_2_ and [H^+^] is altered due to altered buffering capacity, which has implications for how P_a_CO_2_ is transduced into a vasodilatory stimulus (Hoiland, Fisher, & Ainslie, 2019). The magnitude of change in CBF in hypoxia is related to four reflex mechanisms factors when CO_2_ is uncontrolled: (I) hypoxic ventilatory response; (II) hypercapnic ventilatory response at rest; (III) hypoxic cerebral vasodilation; and (IV) hypocapnic cerebral vasoconstriction (Brugniaux et al., 2007). CVR in hypoxia is still unclear as controversial results were obtained: CVR in hypoxia was increased during hyperoxic poikilocapnia (Fan et al., 2010) and hyperoxic isocapnia (Subudhi, Panerai, & Roach, 2010); decreased during hyperoxic poikilocapnia or unchanged during hypoxic poikilocapnia (Ainslie & Burgess, 2008) and uncontrolled hypercapnia (Jansen, Krins, & Basnyat, 1999). To our knowledge, no study has investigated the putative effect of hypobaria on CVR when exposed to acute hypoxia (i.e., NH vs. HH).

The present study adds novelty by also evaluating CVR in a hypobaric normoxic (HN) condition. Isolating the hypobaric effect from the hypoxic one would allow comparing similar normoxic conditions with different barometric pressures (P_B_). The HN condition is when low P_B_ is combined with hyperoxic breathing to obtain an inspired pressure of oxygen (P_I_O_2_) similar to normobaric normoxia (NN). When exposed to hypobaria, the air density is reduced (Conkin, 2016), which may reduce air flow resistance and work of breathing (Loeppky et al., 1997; Ogawa, Fujii, Kurimoto, & Nishiyasu, 2019).This may lead to change in ventilatory pattern (i.e., increased maximal ventilation in HN compared to NN) (Ogawa et al., 2019). Moreover, it was suggested that the ventilatory dead space is increased by hypobaria in hypoxia (Savourey et al., 2003) and normoxia (Ogawa et al., 2019), which could underlie the reported differences in the ventilatory and blood gas parameters. When dead space is greater, P_ET_CO_2_‐P_a_CO_2_ gradient may be increased (Donnellan, 2011). Decrease in barometric pressure has been reported to also increase pulmonary vascular vasoconstriction pressure due to the lower air density in hypobaria (Conkin, 2016). More precisely, pulmonary resistance was increased in hypobaria (HN and HH), independent of oxygen tension, suggesting that pulmonary blood flow may be changed in hypobaria (Petrassi et al., 2018). Moreover, different fluid and acid–base balance responses mediated by augmentation of aldosterone and altered cell‐membrane permeability have been suggested as a consequence of hypobaria (Loeppky et al., 2005). Nevertheless, the effects of hypobaria on the ventilatory responses and CVR responses using HN conditions are scarcely explored and to our knowledge, there is no study comparing CVR in NN versus HN and NH versus HH conditions.

The implications of CVR in hypobaric normoxia/hypoxia are therefore of interest in the context of both aviation (pilots and passengers) and high‐altitude residents/mountaineers/workers, as these populations may be exposed to hypobaric environment with supplemental oxygen. In the present study, we aimed to evaluate the putative effect of hypobaria during acute exposure between conditions with comparable P_I_O_2_ (NH vs. HH and NN vs. HN) on CVR. We also aimed to investigate the hypoxic effect on CVR for conditions with same P_B_ (NN vs. NH and HN vs. HH). We hypothesized that acute hypoxic exposure would induce a left shift and increase in CVR, which would be more exaggerated in hypobaria. This CVR regulation would be effective for maintaining cDO_2_ in all conditions.

## 2 MATERIALS AND METHODS

### 2.1 Ethical approval

This study was performed according to the Declaration of Helsinki and was approved by the Swiss Ethic Committee of Zürich (Swissethics, BASEC ID: 2017–00752). This clinical trial can be found on ClinicalTrials.gov (ID: NCT03303118). All participants were informed about all procedures of this study and gave their written informed consent before participating to this study.

### 2.2 Subject recruitment and screening

Nine healthy pilot trainees (seven men and two women, age 28 ± 4 years; height 176 ± 5 cm; weight 70 ± 10 kg) participated voluntarily in this study. None of the participant was exposed to hypoxia before enrolment in the present study and/or to altitude in the days preceding the trials. A physician screened the participants during a familiarization visit to ensure they were healthy and did not report any medical‐ or altitude‐related issues. Moreover, none of the participants was on medication during the present study. After obtaining written informed consent, participants were enrolled and took part to the test visit.

### 2.3 Study design

This study was conducted at the Aeromedical Center (AeMC), medical center of the Swiss Air Force, in Dübendorf in Switzerland. Participants came for a test visit and underwent experimental trials at sea level (Dübendorf, 440 m, P_B_: 726 ± 5 mmHg) and hypobaric and/or hypoxic conditions. Material was first installed on the subjects, and then participants underwent a pre‐test in normobaric normoxia (Pre‐). In a randomized order, participants undertook four experimental conditions of 30 min (3,000 m HH; 5,500 m HH; NH to simulate 5,500 m of altitude and 5,500 m HN) in the Swiss army hypobaric chamber interspersed with three periods of 30 min in normoxia for total session duration of 5 hr. Twenty‐four hours before all visits, participants were asked to avoid physical exercise, heavy meal, and alcohol or caffeine consumption. Participants remained at rest, seated, during the entire experimental procedures. Each period consisted of (a) 5 min of acclimatization; (b) capillary blood gas sample; (c) 7 min seated at rest with eyes closed for electroencephalography and heart rate variability measurement; (d) 4 min to assess a cognitive test; and (e) hypercapnic modified breathing test. The hypercapnic modified breathing test was performed after 20 min of condition exposure.

### 2.4 Conditions comparison

To evaluate putative hypobaric effect between normoxic and hypoxic conditions, P_I_O_2_ between NN versus HN (141 ± 1 vs. 133 ± 3 mmHg) and NH versus HH (74 ± 1 vs. 70 ± 2 mmHg) were compared by adjusting P_B_ in the hypobaric chamber or F_I_O_2_ based on known equation (P_I_O_2_ = (P_B_‐47) × F_I_O_2_), when 47 mmHg corresponds to water vapor pressure at 37°c (Conkin, 2016). Participants breathed ≈11% and ≈40% O_2_ gas mixture (0.03% CO_2_) concentration for NH and HN, respectively, while P_B_ remained similar between NH and NN, but was decreased similarly in HN and HH.

### 2.5 Experimental procedure

#### 2.5.1 Modified hypercapnic breathing

Participants wore a mask and breathed through a two‐way Y‐valve, which allowed switching from ambient air in the hypobaric chamber to a hermetic bag filled with a hypercapnic gas mixture (20.9% O_2_, 5% CO_2_). For NH and HN conditions, participants were switched from a first gas mixture (≈11% O_2_, 0.03% CO_2_ or ≈40% O_2_, 0.03% CO_2_ respectively) to the hypercapnic gas mixture (respectively ≈11% O_2_, 5% CO_2_ or ≈40% O_2_, 5% CO_2_). As a baseline before hypercapnia, participants were asked to hyperventilate for 1 min to lower their end‐tidal partial pressure of CO_2_ (P_ET_CO_2_). This over‐breathing period was sufficient to induce the same level of P_ET_CO_2_ than with 3 min in a previous study (~18 mmHg at 5,260 m; Fan et al. 2016). Then, subjects breathed normally for 30 s and were switched to the hypercapnic mixed gas for 3 min. Participants were instructed to breathe ad libitum. After completing the hypercapnic breathing test, subjects were finally switched back to the initial gas mixture.

#### 2.5.2 Pulse oxygen saturation

Earlobe pulse oxygen saturation was monitored using an oximeter (3100 pulse oximeter, Nonin) and acquired at 0.5 Hz.

#### 2.5.3 Cerebral blood flow velocity

Middle cerebral artery velocity (MCAv, an index of cerebral blood flow) was measured in the left middle cerebral artery using a 2‐MHz pulsed Doppler ultrasound system (ST3, Spencer technology). The Doppler ultrasound probe was positioned over the left temporal window and held in place with an adjustable plastic headband (Marc 600 Headframe, Spencer technology). The signal was acquired at depths ranging from 43 to 54 mm. Signal quality was optimized and basal MCAv characteristics were recorded to facilitate subsequent probe placements.

#### 2.5.4 Respiratory variables

Gas exchanges data were recorded using a gas analyzer (K5, Cosmed) that was calibrated outside of the hypobaric chamber before each session. Flow volume was calibrated with a 3L syringe. After calibrating zero CO_2_ with scrubber, reference gas was assessed using a certified Cosmed gas concentration (16% O_2_ and 5% CO_2_). Ventilatory data were recorded by the analyzer and exported in Cosmed software for later analysis (OMNIA, Cosmed, Roma, Italy).

#### 2.5.5 Cerebral oxygen delivery

Cerebral oxygen delivery (cDO_2_) was calculated based on MCAv and estimated arterial oxygen content (CaO_2_) with known equation (cDO_2_ = MCAv_mean_ × CaO_2_). CaO_2_ can be estimated with hemoglobin concentration ([Hb]) and pulse oxygen saturation (SpO_2_) values with following equation (CaO_2_ = [Hb] × 1.36 × SpO_2_/100). [Hb] was measured with same device as blood gases described above. cDO_2_ was estimated in each conditions for three periods: (a) last 30‐s baseline, (b) last 30‐s hyperventilation, and (c) last 30‐s hypercapnic gas breathing.

#### 2.5.6 Capillary blood gases

Capillary blood samples were taken at rest on distal part of a finger at the end of the acclimatization phase (i.e., 5 min after exposure). After cleaning up with alcohol, finger extremity was pitched using a lancet and blood sample was acquired in a capillary tube. Following standardized calibration, all blood samples were directly analyzed with a capillary blood‐gas analyzing system (OPTI CCA‐TS, OPTI Medical Systems, Roswell, GA, USA) for capillary blood parameters: Hemoglobin concentration ([Hb]); capillary O_2_ saturation (SO_2_, %); pH; partial pressure of capillary O_2_ (PO_2_); and CO_2_ (PCO_2_).

### 2.6 Data analysis

#### 2.6.1 Cerebrovascular CO_2_ reactivity analysis

**Figure 1:**
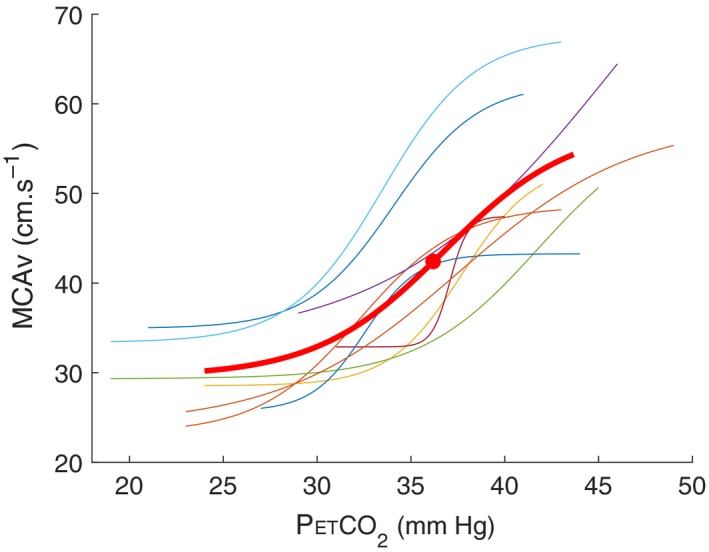
A representative example of sigmoidal curves of all subjects (*n* = 9, i.e., in colors) with mean value (bold curve) during hypercapnic test in normobaric normoxia (NN, Dübendorf 440 m). Bold point represents midpoint

Individual fit of each sigmoid curve and the associate parameters (i.e., midpoint and slope) were calculated (Figure 1). Representing CVR using a sigmoid model allows the determination of a midpoint, which corresponds to the optimal operating point of vessels capacity to dilate and constrict (i.e., reserve of cerebral vessels) (Fan et al., 2016). Previous studies have fitted CVR using a sigmoid model (Ainslie & Duffin, 2009; Fan et al., 2016). Some physiological parameters may be missed using a linear model: optimal operating point and physical constraints of the cerebral vasculature (i.e., vascular reserve) (Battisti‐Charbonney, Fisher, & Duffin, 2011). Moreover, CVR is sigmoidal with a linear portion between PaCO_2_ of 25–65 mmHg under constant arterial blood pressure (Madden, 1993). For these reasons, a sigmoidal model was used for CVR analysis in the present study. Midpoint is the middle between minimal and maximal values when the range of P_ET_CO_2_ is large enough to elicit maximal vasodilatory response. However, the midpoint is also the inflexion point (i.e., where the slope is maximal). It is found where the first derivative is maximal. In this study, the max slope in all cases was detected using the first derivative (independently of the min and max values). If P_ET_CO_2_ elicited the minimal and maximal values of the sigmoid shape, the midpoint would not have changed. The maximum slope of the sigmoid curve is a reasonable assumption for CO_2_ sensitivity (Ainslie & Duffin, 2009; Fan et al., 2016). In a sigmoidal curve, the maximum slope is found at the inflexion point, which is also the midpoint. It is found at the maximum of the derivative. As the slope increases, CVR is more sensitive (i.e., greater capacity to constrict and dilate), but in a smaller range of P_ET_CO_2._

### 2.7 Statistical analysis

One‐way repeated measures ANOVA was assessed for all parameters (SpO_2_, MCAv, P_ET_O_2,_ P_ET_CO_2_, and cDO_2_ absolute values) to test significance between altitude level (NN, 3,000 m and 5,500 m in HH) and each conditions (NN, 5,500 m HH, NH, and HN) using Jamovi software (Jamovi project (2018, version 0.9). Statistical analysis for sigmoid parameters (midpoint and slope) using mixed model (R, R Foundation for Statistical Computing). Significant difference was set for *p* < .05.

## 3 RESULTS

### 3.1 Hypoxic effect in hypobaric hypoxia

**Figure 2:**
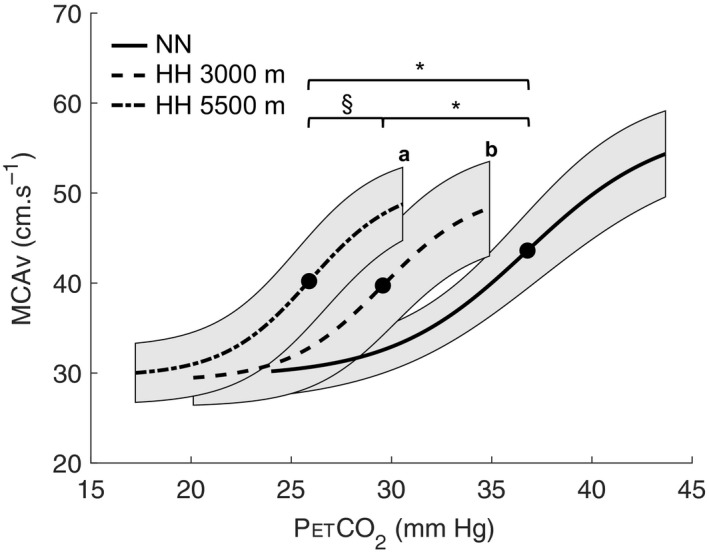
Mean sigmoidal curves of all subjects (*n* = 9): In normobaric normoxia (NN, Dübendorf 440 m); 3,000 m and 5,500 m in hypobaric hypoxia (HH) conditions. Bold point represents midpoint. *p < .05 midpoint different than NN; ^§^ *p* < .05 midpoint different than 3,000 m; (a) *p* < .05 slope different between 5,500 m and NN; (b) *p* < .05 slope different between 3,000 m and NN. Shaded areas surrounding the sigmoid curves represent the 95% confidence interval

There was a significant increase in CVR with increased altitude levels (Figure 2) in HH conditions. Data of the sigmoid curves for each condition are represented in Table 1. Midpoint was significantly lowered at 3,000 m (27.3 ± 2.0 mmHg) and 5,500 m (19.6 ± 2.0 mmHg), compared to NN (35.7 ± 3.3 mmHg, *p* < .001). Midpoint was decreased at 5,500 m compared to 3,000 m (*p* < .001). Compared to NN (0.23 ± 0.12), the slope of sigmoid curve was significantly increased at 3,000 m (0.52 ± 0.27, *p* = .007) and 5,500 m (0.66 ± 0.33, *p* < .001) in HH. However, there was no significant change in slope between 3,000 m and 5,500 m HH.

**Table 1:** Absolute values are means** ±** *SD* (*n* = 9). Mean sigmoidal curve data: Midpoint (mmHg) and inclination (slope) of the sigmoid curve

	NN	HH 3000 m	HN	NH	HH 5500 m
Midpoint	35.7 ± 3.3	27.3 ± 2.0*	21.6 ± 1.9*	33.7 ± 1.7#	19.6 ± 2.0*§†
Slope	0.23 ± 0.12	0.52 ± 0.27*	0.46 ± 0.12(*)	0.35 ± 0.19	0.66 ± 0.33*†

Note

In normobaric normoxia (NN, Dübendorf altitude level of 440 m), hypobaric hypoxia (HH, at altitude level of 3,000 m and 5,500 m), hypobaric normoxia (HN, altitude level of 5,500 m in normoxia), and normobaric hypoxia (NH, altitude simulation of 5,500 m in normobaria). Statistical analysis was performed separately for altitude comparison in HH (NN, 3,000 m and 5,500 m HH) and for conditions comparison (NN, HN, NH, and 5,500 m HH). (*) *p* = .069, *p<.05 different from NN conditions; §*p* < .05 different from 3,000 m HH; #*p* < .05 different from HN; and †*p* < .05 different from NH.

SpO_2_ and MCAv are represented in Table 2. SpO_2_ during baseline was lower in 5,500 m HH than 3,000 m HH and NN (*p* < .001). MCAv during baseline was increased in 5,500 m HH when compared to NN and 3,000 m HH (*p* < .001).

**Table 2:** Absolute values are means** ±** *SD* (*n* = 9)

	Period	NN	HH 3000 m	HN	NH	HH 5500 m
SpO_2_ (%)	Baseline	99.3 ± 1.0	93.5 ± 3.7 (*)	98.2 ± 2.0	80.9 ± 5.2*#	78.1 ± 8.7*§#
	Hyperventilation	99.7 ± 0.6	98.6 ± 1.3	99.1 ± 1.5	94.0 ± 4.4*(#)	92.6 ± 5.5*§#
	Hypercapnia	99.6 ± 0.7	96.4 ± 3.4	98.6 ± 2.1	90.3 ± 5.2*#	85.5 ± 5.5*§#
MCAv (cm/s)	Baseline	45.7 ± 7.9	43.8 ± 9.9	47.0 ± 9.2	50.0 ± 8.2	51.6 ± 11.8*§
	Hyperventilation	29.7 ± 4.5	29.9 ± 5.6	31.5 ± 5.5	34.4 ± 7.2	33.1 ± 6.1*(§)
	Hypercapnia	52.5 ± 8.0	47.1 ± 9.1	50.0 ± 11.5	55.4 ± 7.3	49.4 ± 7.7

Note

Pulse oxygen saturation (SpO_2_), middle cerebral artery velocity (MCAv), minute ventilation (VE), breathing frequency (BF), tidal volume (VT), end‐tidal pressure in carbon dioxide (P_ET_CO_2_) and oxygen (P_ET_O_2_). For time period: baseline, hyperventilation, and hypercapnia (5% CO_2_). In normobaric normoxia (NN, Dübendorf altitude level of 440 m), hypobaric hypoxia (HH, at altitude level of 3,000 m and 5,500 m), hypobaric normoxia (HN, altitude level of 5,500 m in normoxia), and normobaric hypoxia (NH, altitude simulation of 5,500 m in normobaria). Statistical analysis was performed separately for altitude comparison in HH (NN, 3,000 m and 5,500 m HH) and for conditions comparison (NN, HN, NH, and 5,500 m HH). (*) *p* = .081, *p < .05 different from NN conditions; (§) *p* = .053, §*p* < .05 different from 3,000 m HH; (#) *p* = .069, #*p* < .05 different from HN. No significant difference between conditions with comparable P_I_O_2_: NH versus HH and NN versus HN.

Ventilatory data are presented in Table 3. Minute ventilation resting values were increased in 5,500 m HH (16.0 ± 2.7 L/min) compared to all other conditions. P_ET_O_2_ and P_ET_CO_2_ were decreased in HH conditions compared to NN, with lower values at 5,500 m when compared to 3,000 m during baseline.

**Table 3:** Absolute values are means ± *SD* (*n* = 9)

	Period	NN	HH 3000 m	HN	NH	HH 5500 m
V_E_ (L/min)	Baseline	12.1 ± 1.4	12.5 ± 1.4	10.3 ± 1.4	12.1 ± 2.7	16.0 ± 2.7*§#†
	Hyperventilation	39.5 ± 7.7	35.0 ± 8.0	35.6 ± 9.2	35.4 ± 6.9	40.4 ± 10.5†
	Hypercapnia	15.2 ± 4.4	13.6 ± 3.0	11.4 ± 2.0	17.8 ± 4.0#	14.1 ± 2.9†
BF (cycle/min)	Baseline	15.9 ± 2.6	16.7 ± 2.8*	17.9 ± 3.0	17.0 ± 3.6	17.9 ± 2.7*
	Hyperventilation	12.7 ± 3.9	12.0 ± 2.9	12.0 ± 2.5	15.0 ± 4.8	11.5 ± 1.5
	Hypercapnia	16.1 ± 2.4	16.4 ± 2.1	17.2 ± 2.8	17.3 ± 2.8	16.0 ± 3.4
VT (L)	Baseline	0.82 ± 0.21	0.79 ± 0.20	0.62 ± 0.21	0.79 ± 0.27	0.98 ± 0.31 (*)§
	Hyperventilation	3.35 ± 0.99	3.04 ± 0.90	3.12 ± 1.02	2.52 ± 1.00*	3.62 ± 0.80§†
	Hypercapnia	1.04 ± 0.23	0.84 ± 0.17*	0.68 ± 0.16*	1.06 ± 0.24#	0.93 ± 0.26 (†)
P_ET_O_2_ (mmHg)	Baseline	99.4 ± 8.0	59.7 ± 6.7*	85.3 ± 9.4*	45.0 ± 3.3*#	36.1 ± 4.7*§#
	Hyperventilation	125.8 ± 4.7	81.4 ± 5.4*	102.6 ± 12.7*	58.9 ± 9.1	50.0 ± 7.1*§#
	Hypercapnia	117.2 ± 7.1	70.0 ± 5.8*	86.8 ± 12.9*	58.6 ± 6.2*#	41.4 ± 4.5*§#†
P_ET_CO_2_ (mmHg)	Baseline	34.5 ± 2.8	28.5 ± 2.5*	22.3 ± 2.4*	30.8 ± 2.2*#	19.9 ± 1.3*§†
	Hyperventilation	24.0 ± 3.9	20.8 ± 3.0	17.0 ± 3.2*	24.2 ± 4.3#	15.5 ± 2.6*§†
	Hypercapnia	42.0 ± 2.8	31.4 ± 3.3*	25.1 ± 1.7*	40.5 ± 2.1#	22.1 ± 1.7*§#†

Note

Ventilatory parameters: Minute ventilation (VE), breathing frequency (BF), tidal volume (VT), end‐tidal pressure in oxygen (P_ET_O_2_) and carbon dioxide (P_ET_CO_2_). For time period: baseline, hyperventilation, and hypercapnia (5% CO_2_). In normobaric normoxia (NN, Dübendorf altitude level of 440 m), hypobaric hypoxia (HH, at altitude level of 3,000 m and 5,500 m), hypobaric normoxia (HN, altitude level of 5,500 m in normoxia), and normobaric hypoxia (NH, altitude simulation of 5,500 m in normobaria). Statistical analysis was performed separately for altitude comparison in HH (NN, 3,000 m and 5,500 m HH) and for conditions comparison (NN, HN, NH, and 5,500 m HH). (*) *p* = .061, *p < .05 different from NN conditions; §*p* < .05 different from 3,000 m HH; #*p* < .05 different from HN; and (†) *p* = .058, †*p* < .05 different from NH.

cDO_2_ values during CVR assessment (for baseline, hyperventilation, and hypercapnia periods) are displayed in Figure 4. cDO_2_ absolute value was similar during baseline period in NN with HH conditions (3,000 m and 5,500 m).

Capillary blood samples data are shown in Table 4. SO_2_ gradually decreased at 3,000 m (87.9 ± 1.6%) and 5,500 m (75.0 ± 4.0%) in HH when compared to NN (95.3 ± 1.1%, *p* < .001) after 5 min of condition exposure.

**Table 4:** Absolute values are means ± *SD* (*n* = 9). Capillary blood data for hemoglobin concentration ([Hb], g/dl); capillary oxygen saturation (SO_2_, %); capillary blood pH; partial pressure of capillary O_2_ (PO_2_) and CO_2_ (PCO_2_). In normobaric normoxia (NN, Dübendorf altitude level of 440 m), hypobaric hypoxia (HH, at altitude level of 3,000 m and 5,500 m), hypobaric normoxia (HN, altitude level of 5,500 m in normoxia), and normobaric hypoxia (NH, altitude simulation of 5,500 m in normobaria). Statistical analysis was performed separately for altitude comparison in HH (NN, 3,000 m and 5,500 m HH) and for conditions comparison (NN, HN, NH and 5,500 m HH)

	NN	HH 3000 m	HN	NH	HH 5500 m
[Hb] (g/dl)	16.2 ± 1.9	16.9 ± 2.0	16.4 ± 1.4	16.5 ± 1.9	17.1 ± 1.6
SO_2_ (%)	95.3 ± 1.1	87.9 ± 1.6*	92.1 ± 2.4	81.1 ± 4.0*#	75.0 ± 4.0*§#†
PO_2_ (mmHg)	77.0 ± 3.9	50.9 ± 2.2*	57.0 ± 4.5*	45.0 ± 4.7*#	34.1 ± 2.5*§#†
PCO_2_ (mmHg)	36.2 ± 2.0	29.4 ± 2.8*	30.3 ± 4.2*	35.0 ± 2.7#	24.4 ± 2.2*§#†
pH	7.460 ± 0.015	7.513 ± 0.037*	7.515 ± 0.037*	7.475 ± 0.013#	7.580 ± 0.023*§#†
Hct (%)	48.7 ± 5.8	50.7 ± 6.1	49.1 ± 4.4	49.5 ± 5.8	51.6 ± 5.0

Note

*p < .05 different from NN conditions; §*p* < .05 different from 3,000 m HH; #*p* < .05 different from HN; and †*p* < .05 different from NH.

### 3.2 Hypobaric effect

**Figure 3:**
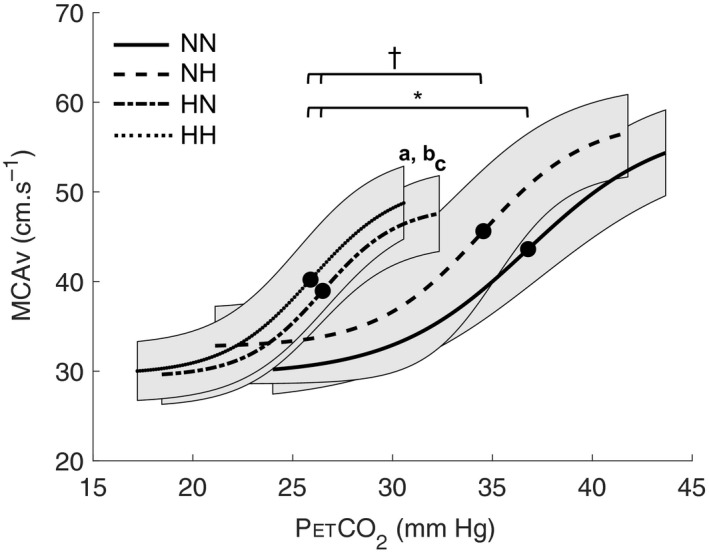
Mean sigmoidal curves of all subjects (*n* = 9) in: normobaric normoxia (NN); normobaric hypoxia (NH); hypobaric hypoxia (HH); and hypobaric normoxia (HN) conditions. Bold point represents midpoint. ^†^ *p* < .05 midpoint different between HH/HN and NH; *p < .05 midpoint different between HH/HN and NN; (a) *p* < .05 slope different between 5,500 m HH and NN; (b) *p* < .05 slope different between 5,500 m HH and NH; (c) *p* = .069 slope tend to be different between HN and NN. Shaded areas surrounding the sigmoid curves represent the 95% confidence interval

There was a decrease in midpoint (left shift) with decreased barometric pressure (Figure 3). Midpoint was significantly lower in 5,500 m HH and HN (21.6 ± 1.9 mmHg), when compared to NN (*p* < .001). Slope was increased in HH compared to normobaric conditions in NH (0.35 ± 0.19, *p* = .003) and NN (*p* < .001). Slope did not change with hypoxia for the same barometric pressure values, when comparing NN versus NH and HH versus HN, respectively. In normoxia, slope in HN tends to be increased when compared to NN (*p* = .069).

During baseline and hypercapnia, SpO_2_ was decreased in hypoxic conditions (NH and 5,500 m HH) when compared to normoxic conditions (NN and HN). MCAv elevation between hyperventilation and the end of hypercapnia (i.e., relative delta, %Δ) tended to be lower in 5,500 m HH (+50.9 ± 18.5%) and HN (+58.6 ± 20.6%) than NN (+77.5 ± 9.5%, *p* = .065).

**Figure 4:**
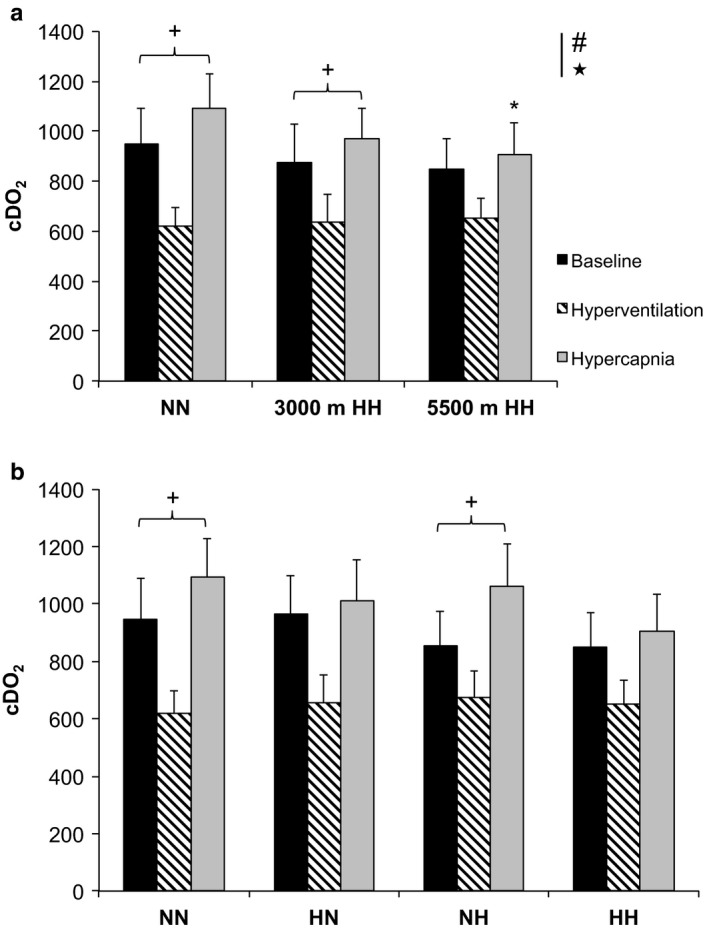
Cerebral oxygen delivery (cDO_2_, absolute values) of all subjects (*n* = 9), Mean** ±** *SD*. (a) Normobaric normoxia (NN) and hypobaric hypoxia (HH) conditions at 3,000 m and 5,500 m. (b) NN; normobaric hypoxia (NH); hypobaric hypoxia (HH), and hypobaric normoxia (HN) conditions. Left histograms represent cDO_2_ baseline values, middle cDO_2_ during hyperventilation, and right cDO_2_ at the end of hypercapnia. ^#^ *p* < .05 for difference between baseline and hyperventilation values in all conditions; ^★^ *p* < .05 for difference between hyperventilation and hypercapnia values in all conditions; ^+^ *p* = .014, ^+^ *p* < .05 for difference with baseline values; and *p = .046 for difference with NN during hypercapnia

cDO_2_ was similar during baseline and decreased to the same extent (*p* < .001) during hyperventilation in all conditions (Figure 4a). Interestingly, cDO_2_ during hypercapnia was higher than baseline values only in the normobaric conditions (NN and NH), but not in the hypobaric conditions (HN and HH, Figure 4b). When compared to NN, cDO_2_ during hypercapnia was decreased in 5,500 m HH (*p* = .046) but not in NH. As participants were in normoxia (i.e., breathing hyperoxic gas mixture) in HN condition, cDO_2_ was similar during hypercapnia between NN and HN. Our data suggest no significant difference in cDO_2_ during hypercapnia between conditions with similar P_I_O_2_ (i.e., NH vs. HH and NN vs. HN).

Capillary blood sample showed a lower SO_2_ (*p* < .001) in NH (81.1 ± 4.0%) and 5,500 m HH (74.0 ± 4.0%) compared to normoxic conditions (NN and HN: 92.1 ± 2.4%). Moreover, SO_2_ was lower in NH than HH (*p* = .013).

## 4 DISCUSSION

In the present study, we investigated cerebrovascular changes during CO_2_ breathing comparing parameters of sigmoid curve in various normobaric versus hypobaric and normoxic versus hypoxic conditions. We also calculated cDO_2_ in all conditions for three successive periods (baseline, hyperventilation, and hypercapnia) during CVR assessment. The main results are as follows: (a) A left shift in P_ET_CO_2_‐MCAv sigmoid curve with an increase in CVR with altitude level in HH. The same observation was observed under the influence of hypobaria for a similar P_I_O_2_ (i.e., significant for HH vs. NH and a trend for HN vs. NN). We observed also an influence of hypobaria per se on CVR, mediated by hypocapnia (i.e., sigmoid midpoint left‐shift); (b) No hypoxic effect on CVR for equivalent barometric pressure (NN vs. NH) and (HN vs. HH); and (c) cDO_2_ was maintained during baseline in all conditions, but the cerebrovascular reserve was reduced in the hypobaric conditions (HH and HN) compared to the normobaric ones (NN and NH). This resulted in decreased cDO_2_ in 5,500 m HH condition during hypercapnia.

### 4.1 Increased cerebrovascular reactivity to CO_2_ in hypobaria

Under hypoxia, hyperventilation‐induced hypocapnia is accelerated by an increase in peripheral respiratory chemoreflex (Ogoh, 2019). Moreover, it has been previously shown that there were greater hypocapnia and blood alkalosis when exposed to HH than NH (Savourey et al., 2003). To our knowledge, there is no study comparing the CVR during acute exposure in HH versus NH. The present results showed a left shift in CVR sigmoid curve in HH, in line with a previous study at high altitude while breathing hyperoxic mixed gas (Fan et al., 2010). Many studies have evaluated the cerebrovascular reactivity to CO_2_ in humans exposed to high altitude (Ainslie & Burgess, 2008; Fan et al., 2016, 2010; Flück, Siebenmann, Keiser, Cathomen, & Lundby, 2015; Jansen et al., 1999; Jensen et al., 1996; Lucas et al., 2011; Willie et al., 2015). However, CVR in hypoxia remains unclear with controversial results. For instance, CVR in hypoxia was during hyperoxic poikilocapnia (Fan et al., 2010) and hyperoxic isocapnia (Subudhi et al., 2010); decreased during hyperoxic poikilocapnia (Ainslie & Burgess, 2008) or unchanged during hypoxic poikilocapnia (Ainslie & Burgess, 2008) and uncontrolled hypercapnia (Jansen et al., 1999). Nevertheless, it is known that CBF response to CO_2_ is blunted in hypoxia by potentially limiting dilatory responses (Fan, Bourdillon, & Kayser, 2013; Leffler, Busija, Beasley, Fletcher, & Green, 1986; McPherson, Eimerl, & Traystman, 1987). In the present study, the slope of the sigmoid curve was significantly increased in 5,500 m HH compared to NH, suggesting a specific effect of hypobaria on CBF response to CO_2_ when exposed to hypoxia. Reduced reactivity results in less central CO_2_ washout and greater ventilatory stimulus (Peebles et al., 2007). In fact, minute ventilation was greater in NH than HH during hypercapnia, whereas reactivity was increased in HH in the present study. However, due to its interaction with hypoxia, cerebrovascular responses to CO_2_ in such environment should be interpreted with caution. In hypoxia, CVR may not reflect true vasoreactivity (Fan et al., 2016), as CVR may be affected by the hypoxia‐induced vasodilation (Gupta, Menon, Czosnyka, Smielewski, & Jones, 1997).

In the present study, capillary blood samples showed a lower SO_2_ in 5,500 m HH than NH after 5 min of condition exposure, which is in line with the larger hypoxemia observed in HH than NH (Savourey et al., 2003). However, these values should be evaluated with cautious, as capillary blood sample was not measured during the peak ventilatory response that occurs during the first 2 min of poikilocapnic hypoxia exposure (Steinback & Poulin, 2007). Interestingly, SO_2_ (at 5 min) showed a difference between NH and HH of ~ 6% while SpO_2_ difference (measured at 20 min during CVR) was 2%–3% during baseline of the hypercapnic test (Table 2). Collectively, these results support that hypoxemia may influence CVR. However, because of temporal dissociation between measurements, blood gas values (shown in Table 4) were not use to discuss CVR differences. MCAv resting values during baseline were only significantly increased in 5,500 m HH when compared to NN and 3,000 m HH (Table 2). MCAv was logically decreased during hyperventilation due to the hypocapnia‐induced vasoconstriction (Kaur et al., 2018). Then, hypercapnia triggers cerebral vasodilation, which induces an increase in MCAv. MCAv increases during hypercapnia to wash out CO_2_ from the brain tissue to regulate and maintain cerebrospinal fluid pH (Xie et al., 2006). In hypobaric conditions (i.e., HH and HN), CVR showed a left shift of the midpoint, indicating a resetting to a lower P_ET_CO_2_ values (i.e., hypocapnia). On the contrary, NH induced smaller left shift compared to HH, likely due to a lesser hyperventilation. Consequently, our results indicate an effect of hypobaria per se on CBF, as we observed significant differences between NH versus HH regarding midpoint and the slope of the sigmoid curve.

The analysis of CO_2_ sensitivity is based on the subjects’ exposure to a range of arterial CO_2_ going from hypocapnia to hypercapnia. In the present study, hypocapnia resulted from voluntary hyperventilation of the subjects (as instructed by the experimenters in the present study and in a previous study (Fan et al., 2016)). Then, the subjects breathed normally so that capnia went back to initial value, at which point the subjects were exposed to 5% CO_2_ to create the hypercapnic exposure. With such methods, we had P_ET_CO_2_ values ranging from 15.5 ± 2.6 to 42.0 ± 2.8 mmHg (i.e., from hyperventilation to hypercapnia in 5,500 m HH and NN conditions, respectively) which is comparable to rebreathing methods although with slightly less progressive changes in the hypercapnic part (Ainslie & Duffin, 2009; Fan et al., 2016). However, the sigmoid behavior of the CO_2_ response could clearly be seen, as expected and the fits were of good quality (Figure 1).

We also evaluated CVR in acute HN environment to determine the putative influence of hypobaria on CVR in normoxia. Some early studies have explored the effects of hypobaric normoxic (HN), when exposed to chronic high altitude while breathing pure enriched oxygen gas mixture (Cerretelli, 1976; Marconi et al., 2004). These studies reported higher $\dot{V}$O_2max_ value in HN than in NN and postulated that it might arise from a lower air density. Similarly, $\dot{V}$E_max_ was reported to be higher and the time to exhaustion during incremental running on treadmill to be extended under HN compared to NN, showing an enhanced exercise performance in HN, when air density is reduced (Ogawa et al., 2019). In the present study, cerebrovascular reactivity to CO_2_ was assessed under hypobaric conditions (HH and HN). Our results showed a left shift of midpoint in HH and HN compared to NN, indicating a specific hypobaric effect on CVR. The influence of barometric pressure on respiratory pattern (lower tidal volume and higher breathing frequency) was observed in hypoxia (HH vs. NH) (Savourey et al., 2003). In hypobaria, the putative increased physiological dead space and altered alveolo‐capillary diffusion in HH compared to NH (Millet et al., 2012). The present results of $\dot{V}$E (10.3 vs. 12.1 L/min in HN vs. NN) are in line with previous values in HN versus NN at rest (11.5 vs. 15.6 L/min) (Petrassi et al., 2018). The lower P_ET_CO_2_ in HN versus NN was observed in the three phases (baseline, hyperventilation, and hypercapnia) without any hyperventilation. First, one cannot rule out that the inhalation of hyperoxic gas mixture (F_I_O_2 _~40%) needed in HN for normalizing P_I_O_2_ may have a direct (yet unclear) effect on ventilation and P_ET_CO_2_. Second, the increased dead space in hypobaria has an influence on P_ET_CO_2_‐PaCO_2_ gradient. When dead space is greater, P_ET_CO_2_‐PaCO_2_ gradient may be increased (Donnellan, 2011). The present data of the decoupling between P_ET_CO_2_ and $\dot{V}E$ between HN and NN (i.e., decreased P_ET_CO_2_ without increased $\dot{V}$E in the present study at rest) was already observed (Ogawa et al., 2019) at maximal intensity (i.e., increased $\dot{V}$E without decreased P_ET_CO_2_). This last observation suggests a complex interaction between hypobaria and hypoxia on ventilatory responses. The mechanisms remain unclear and deserve further investigation on these specific ventilatory responses (the present study focusing more on CVR).

### 4.2 Relation between cerebrovascular reactivity and hypocapnia

A recent review on cerebrovascular reactivity discussed the importance of change in PaCO_2_ as a mediator of cerebral microvascular hemodynamic function (Ogoh, 2019). It is mentioned that there is a decrease or increase in MCAv induced by cerebral constriction or dilation, when PaCO_2_ is low or high (i.e., hypo‐ or hypercapnia, respectively) (Markwalder, Grolimund, Seiler, Roth, & Aaslid, 1984). In addition, it was shown that cerebral autoregulation also is enhanced or attenuated by hypocapnia or hypercapnia, respectively (Aaslid, Lindegaard, Sorteberg, & Nornes, 1989). More specifically, full restoration of blood flow to the pretest level was seen in hypocapnia (i.e., after 4.1 s), while the response was slower in normo‐ and hypercapnia (Aaslid et al., 1989). Thus, it is likely that changes in PaCO_2_ may influence the myogenic tone of cerebral vasculature and affect the dynamic of cerebral autoregulation (Ogoh, 2019). However, it appeared that there is a close relationship between extracellular pH and the contractile response of cerebral arteries and arterioles, independently of PCO_2_ (Kontos, Raper, & Patterson, 1977; Toda, Hatano, & Mori, 1989). In the present study, CVR was increased in HH conditions (i.e., greater sigmoid slope), when hypocapnia and increased minute ventilation was observed. During acute hypoxic exposure, respiratory alkalosis is observed as a [HCO_3_^−^] reduction in cerebrospinal fluid, leading to a greater elevation in [H^+^] for a given increase in PCO_2_ (Siesjö, 1972). Moreover, the sigmoid slope remained increased in acute high‐altitude exposure when plotting MCAv against [H^+^] (Fan et al., 2016), suggesting that cerebrovascular reactivity to CO_2_ was likely mediated by an increase in [H^+^] sensitivity (Fan et al., 2016). As [H^+^] was not measured during hypercapnic procedure in the present study, we have plotted the sigmoid slope against P_ET_CO_2_ only.

One may speculate that the increased CVR in acute hypobaric conditions (i.e., HH and HN) may be mediated by the respiratory alkalosis‐induced hypocapnia. On the contrary, minute ventilation remained unchanged with similar CVR and no significant left shift in midpoint (i.e., no hypocapnia) in NH condition compared to NN. Therefore, our results indicate a hypobaric effect on cerebrovascular reactivity to CO_2_ more pronounced between hypoxic than normoxic conditions (i.e., NH vs. HH and NN vs. HN, respectively).

### 4.3 Alteration in cerebrovascular reserve affects the cerebral oxygen delivery in hypobaria

It has been previously shown that cerebrovascular reserve was impaired at high altitude when midpoint was reset to a lower resting arterial PCO_2_ (Fan et al., 2016). Midpoint corresponds to the optimization point of a sigmoid curve between maximal vasoconstriction and vasodilation (Battisti‐Charbonney et al., 2011). Previous study showed lowered resting arterial PCO_2_ by around 12 mmHg on acute exposure to 5,260 m (Subudhi et al., 2014). In the present study, we observed an increase in cerebral oxygen delivery during hypercapnia compared to baseline in normobaric conditions (NN and NH) while cDO_2_ remained similar to baseline values in hypobaric conditions (HN and HH): This suggests a lower vascular dilation capacity (i.e., lower MCAv increase) in hypobaria and suggests that the alteration in cerebrovascular reserve due to hypoxia is higher in hypobaric than in normobaric conditions (Figure 4). Interestingly, the MCAv increase between hyperventilation and the end of hypercapnia (relative delta, %Δ) tended to be lower in 5,500 m HH (+50.9 ± 18.5%) and HN (+58.6 ± 20.6%) than in NN (+77.5 ± 9.5%, *p* = .065). This could explain the decreased cDO_2_ in 5,500 m HH during hypercapnia. Our data suggest that the vasodilation reserve was diminished in hypobaria (i.e., smaller increase in MCAv from hyperventilation to hypercapnia (%Δ)). Our results indicate a decrement of cerebral blood flow regulation capacity in hypobaric conditions possibly impacting cDO_2_. Our findings support a previous study that showed blunted vessel's ability to respond to change in CO_2_ concomitant to hyperventilation‐induced hypocapnia at high altitude (Fan et al., 2016). Such blunting effect could possibly impair cerebral autoregulation during acute or chronic high‐altitude exposure, as previously demonstrated (Ainslie & Burgess, 2008; Iwasaki et al., 2011; Jansen et al., 1999; Subudhi et al., 2014). We suggest that vascular reserve to dilate may be blunted in hypobaria (HH vs. NH and HN vs. NN, Figure 3), either in hypoxic or normoxic conditions, since midpoint was left shifted. This is of interest since absolute values in cerebral oxygen delivery were similar during baseline and hyperventilation between all conditions. The fact that cDO_2_ was increased during hypercapnia only in normobaric conditions (i.e., NN and NH) when compared to baseline values suggest that this hypobaric effect on cDO_2_ regulation occurs only with hypercapnia. The reliability of the cDO_2_ data is based primarily on three assumptions: (1) MCA diameter is not changing during hypocapnic and hypercapnic states, (2) MCAv represents global CBF, that is, anterior and posterior circulation can be equally represented by just the MCAv; and (3) capillary blood samples provide an accurate index [Hb]. Assumptions 2 and 3 might hold true but assumption 1 likely does not. The present study was designed to discriminate the effects of hypobaria on cerebrovascular reactivity to CO_2_; however, some methodological considerations should be acknowledged when interpreting our findings. Transcranial Doppler ultrasound (TCD) was used to measure MCAv as an index of global CBF changes. This assumed that the MCA carries approximately 80% of the cerebral blood flow to the two hemispheres (Lindegaard et al., 1987); and that the changes in MCAv reflect changes in global CBF (Bishop, Powell, Rutt, & Browse, 1986; Serrador, Picot, Rutt, Shoemaker, & Bondar, 2000).

In addition, on the one hand, the changes in MCAv in response to CO_2_ changes are comparable to the changes in internal carotid blood flow (Sato et al., 2012); and on the other hand, the diameter of the MCA does not change during the observed changes in arterial blood gases (Serrador et al., 2000) or with even stronger stimuli (Fan et al., 2014). In support, MCAv has been shown to reflect changes in CBF assessed with the direct Fick method, at least during initial exposure to high altitude (Milledge, 1979; Møller et al., 2002; Roy et al., 1968). Previous study reported that the MCA diameter remains relatively unchanged up to 5,300 m (Wilson et al., 2011). However, we have not measured the MCA diameter, and it may change (Coverdale, Gati, Opalevych, Perrotta, & Shoemaker, 2014) in the sense that MCAv may overestimate CBF in the hypocapnic and underestimate it in the hypercapnic states. Therefore, calculating CDO_2_ from MCAv during those states may result in smaller differences than those occurring. Hence, potentially explaining why there was no difference in cDO_2_ between conditions (Figure 4b).

Despite alteration in cerebrovascular reserve in HN, cDO_2_ during hypercapnia in HN was not significantly different than in NN. When compared to NN, relative cDO_2_ during hypercapnia was similar in NH but diminished in 5,500 m HH, suggesting a greater influence with hypobaria in hypoxia.

### 4.4 Application in aviation physiology

In the present study, we aimed to be as specific as possible to flight conditions for pilots (i.e., to investigate cerebral responses to CO_2_ as pilots breathing hyperoxic gases at high altitude). Pilots are daily exposed to hypobaric environment during flights either in normoxia (HN) or hypoxia (HH), in case of cabin decompression (Muehlemann, Holper, Wenzel, Wittkowski, & Wolf, 2013) or unpressurized cabins (Nishi, 2011). In addition, military crew may be exposed to hypobaric hypoxic environment during flights, but perform training in flight simulator (i.e., in NH condition). It is thus paramount to investigate how cerebral functions may be altered during acute exposure to various environments, such as NH, HH, and HN conditions.

### 4.5 Limitations

A fixed inspired concentration of CO_2_ was used in the present hypercapnic test, which does not translate to precise control of the actual vasoactive stimulus (i.e., the arterial partial pressure of CO_2_) (Fisher, 2016). Moreover, when breathing a fixed fraction of CO_2_, the gradient between P_ET_CO_2_ (which is measured) and PaCO_2_ (the hemodynamic response determinant) changes, meaning that the representativeness of P_ET_CO_2_ for the stimulus at the arterial level are likely variable (Fisher, 2016). Control of alveolar ventilation through sequential gas delivery should be used in future studies (Fisher, Iscoe, & Duffin, 2016).

Of minor concern is that P_I_O_2_ was not perfectly matched between NN and HN (141 ± 1 vs. 133 ± 3 mmHg), as well as between NH and HH (74 ± 1 vs. 70 ± 2 mmHg) conditions. However, these conditions can still be compared to each other. Based on equation [P_I_O_2_ = F_I_O_2_*(P_B_‐47)] (Conkin, 2016), a difference of 3–4 mmHg in P_I_O_2_ corresponds to approximately 15–20 mmHg of barometric pressure (i.e., 300–400 m of simulated altitude) if inspired oxygen pressure remains stable. During each trial session, barometric pressure in the hypobaric chamber was stabilized (fluctuation of 100–200 m). Meteorology records (by http://www.meteoSwiss.ch) confirmed a variation of 800 m of simulated altitude (between 5,100 m and 5,900 m) for a barometric pressure of 375 mmHg measured at the same location over a year period. Consequently, the difference of 3–5 mmHg of P_I_O_2_ between our experimental conditions in the hypobaric chamber is negligible and much lower than the natural meteorological variability.

## 5 CONCLUSION

The present study was the first one to compare cerebrovascular CO_2_ reactivity during acute exposure in various normobaric/hypobaric and normoxic/hypoxic conditions. The left shift in hypobaric versus normobaric conditions for a similar P_I_O_2_ (i.e., significant in hypoxia for HH vs. NH and a trend in normoxia for HN vs. NN) demonstrates a specific effect of hypobaria on CVR. In hypobaric conditions, CVR showed a left shift of the midpoint, indicating a resetting to a lower P_ET_CO_2_ values. On the contrary, NH induced smaller left shift compared to HH, likely due to a lesser hyperventilation and possibly unaffected P_ET_CO_2_‐PaCO_2_ gradient due to normobaric environment. Our results suggest that vascular reserve to dilate may be blunted in hypobaria (i.e., HH vs. NH and HN vs. NN), either in hypoxic or normoxic conditions, since midpoint was left shifted. This blunt effect in hypobaria could impair cerebral oxygen delivery.

## CONFLICT OF INTEREST

The authors declare no conflict of interest and have no financial relationship to disclose.

## AUTHORS’ CONTRIBUTIONS

MRA, NB, AK, DB, and GPM were part of the conception, protocol design. MRA conducted the experiments, was responsible for data acquisition, and wrote the manuscript. MRA, NB, and GPM interpreted the data. MRA and NB conducted the analysis. NB and GPM revised critically the manuscript and gave advises for corrections to MRA. MRA, NB, AK, DB, and GPM gave their final approval of this version to be published.
